# Coach Candidates’ and Coaches’ Nutrition Knowledge Affects Dietary Recommendations Indirectly: Mediator Effects of Self-Efficacy

**DOI:** 10.3390/nu17030589

**Published:** 2025-02-06

**Authors:** Öykü Altınok, Murat Baş

**Affiliations:** 1 Department of Nutrition and Dietetics, Faculty of Health Science, Fenerbahce University, 34758 Istanbul, Turkey; oyku.altinok@fbu.edu.tr; 2 Department of Nutrition and Dietetics, Faculty of Health Science, Acibadem Mehmet Ali Aydinlar University, 34752 Istanbul, Turkey

**Keywords:** coach, dietary recommendation, nutrition knowledge, nutrition self-efficacy, nutrition counselling self-efficacy, social cognitive theory

## Abstract

**Background/Objectives**: The nutritional knowledge and behavior of athletes are considerably influenced by the dietary recommendations of their coaches, which, in turn, affect their health and performance. In the context of social cognitive theory, this study examines the impact of general and sports nutrition knowledge (GeSNK), nutrition self-efficacy (NSE), and nutrition counseling self-efficacy (NCSE) on the dietary recommendations (DRs) provided by coach candidates and coaches (CC&Cs). Additionally, it explores variations in GeSNK, NSE, and NCSE among CC&Cs based on their sports positions, genders, and types of sports. **Methods**: This study included 70 coach candidates and 102 coaches, with data collected via validated scales for GeSNK, NSE, and NCSE. Using the PROCESS macro, the serial multiple mediator model was applied, and group comparisons were conducted via the Mann–Whitney U test. **Results**: The results revealed that 36% of participants achieved adequate GeSNK scores, but only 6.4% abstained from providing DRs. Participants engaged in individual sports exhibited higher NSE and NCSE scores than those involved in team sports. Additionally, 22.7% of CC&Cs identified their former coaches as a primary source of nutrition information. The study findings revealed that GeSNK, NSE, and NCSE significantly influenced DRs, collectively accounting for 23% of the variance in DRs. The direct effect of NCSE on DRs was statistically significant, whereas the direct effects of GeSNK and NSE were not significant. GeSNK and NSE jointly explained only 41% of the variance in NCSE, suggesting that additional factors influencing NCSE and DRs remain unexplored. **Conclusions**: To address the issue of inadequate dietary recommendations stemming from insufficient information, it is crucial to identify and understand these additional factors affecting NCSE and DRs. Diversifying nutrition education programs to enhance the nutrition knowledge of CC&Cs is essential. Furthermore, fostering collaboration between CC&Cs and nutritionists can ensure that athletes receive accurate and comprehensive nutritional guidance.

## 1. Introduction

Nutrition plays a critical role in reducing injury risk, enhancing training adaptations, optimizing sports performance, and promoting overall health among athletes. Consequently, understanding and improving athletes’ nutritional behaviors has been a longstanding area of interest in sports science [1,2,3,4,5,6,7]. Nutritional behavior encompasses various elements, including food selection, motivation, dietary practices, and challenges related to nutrition [8]. These behaviors are closely related to nutritional knowledge [9,10]. However, research consistently highlights a deficiency in both general and sports-specific nutritional knowledge among athletes, underscoring the need for targeted interventions [1,4,5,7].

Coaches are pivotal sources of nutritional information for athletes [1,2,3,4,5,7,11,12,13,14,15,16,17], particularly in environments where access to nutritionists is limited, such as high school sports teams and fitness centers [15]. Due to their close relationships with athletes and perceived expertise in sports, coaches are often trusted to provide dietary guidance [1]. However, an overreliance on coaches for nutritional advice can constrain athletes’ training outcomes and performance [17].

Many coaches assume roles akin to nutritionists, frequently offering dietary recommendations [14,18] on topics such as hydration, supplement use, weight management, protein intake, and meal timing [14,17,18,19,20,21]. Despite their influence, coaches often lack adequate nutritional knowledge, raising concerns about the accuracy of their advice [1,4,12,13,14,19,22]. Inappropriate dietary recommendations can lead to severe physical and psychological consequences, including the development of eating disorders [23].

Previous studies have examined coaches’ dietary recommendations concerning their nutritional knowledge and experience [21]. However, the psychological factors influencing their behavior remain insufficiently explored. Jacob et al. [16] applied the theory of planned behavior to investigate this issue, identifying subjective norms and perceived behavioral control as key factors affecting coaches’ intentions to provide sports nutrition advice. Improving nutritional knowledge can enhance perceived behavioral control by addressing knowledge barriers and boosting self-efficacy, thereby fostering positive behaviors [15].

Self-efficacy is a fundamental concept in social cognitive theory (SCT), which asserts that behavior, personal characteristics (self-efficacy, outcome expectation, values, etc.), and environmental factors (social models, feedback, standards, rewards, etc.) interact to explain and predict behavioral changes [24,25]. It is frequently used in the literature to clarify human nutrition behavior. Bandura referred to these interactions as “reciprocal determinism”, signifying that alterations in one of the three components will induce changes in the others [26]. Bandura defines self-efficacy as “people’s judgments of their capabilities to organize and execute courses of action required to attain designated types of performances” [24]. Accordingly, nutrition self-efficacy can be defined as a person’s confidence in their ability to perform nutritional behaviors including food selection, motivation, dietary practices, and challenges related to nutrition. Nutrition self-efficacy, in combination with knowledge, significantly influences individuals’ nutritional behaviors [27,28,29,30]. Research shows that while enhancing nutritional knowledge is essential, it must simultaneously bolster self-efficacy to effectively drive behavioral change [31,32]. Nutrition self-efficacy has been identified as a mediating variable between knowledge and behavior [27,31]. Although prior research has assessed coaches’ confidence in their nutritional knowledge [1], their nutrition self-efficacy remains underexplored. Since self-efficacy is influenced by knowledge [32], it is plausible that coaches’ nutritional knowledge directly impacts their self-efficacy. This relationship may subsequently shape athletes’ behaviors through modeling or dietary recommendations, as observing a confident coach can inspire athletes to adopt proper nutrition practices [23,26]. The relationship between coaches’ nutrition self-efficacy and their dietary recommendations is also still unclear.

Another critical factor in this context is nutrition counseling self-efficacy, which is believed to influence coaches’ dietary recommendation behaviors. Nutrition counseling self-efficacy can be defined as a person’s confidence in their ability to provide guidance on others’ nutritional behavior. Similarly to general nutrition self-efficacy, nutrition counseling self-efficacy may be shaped by an individual’s nutritional knowledge. In a specific situation, related self-efficacy constructs are likely to be related to one another. An individual who perceives himself/herself as competent in performing an action is likely to perceive himself/herself as competent in teaching and guiding others. However, since observing a successful performance can increase observers’ self-efficacy [25], coaches and their former coaches can also be a source of nutrition counseling self-efficacy.

While coaches should not be considered substitutes for professional nutritionists, understanding the factors influencing their dietary recommendations is essential for improving athlete outcomes.

In the context of SCT, this study aimed to investigate the effects of general and sports nutrition knowledge (GeSNK), nutrition self-efficacy (NSE), and nutrition counseling self-efficacy (NCSE) on coach candidates and coaches’ (CC&Cs’) dietary recommendations (DRs). Additionally, it examined variations in GeSNK, NSE, and NCSE among CC&Cs based on their gender, sports type, and professional position. For these purposes, answers to the following questions were sought.

1. What are the GeSNK levels, DR frequencies, and nutrition information sources of coaches and coach candidates?
2. Do the GeSNK, NSE, and NCSE scores of coaches and coach candidates show significant differences in terms of (a) gender, (b) sport type, and (c) position?
3. Do GeSNK, NSE, and NCSE directly affect DRs?
4. Do NSE and NCSE mediate the relationship between GeSNK and DRs?

## 2. Materials and Methods

### 2.1. Study Design

**Figure 1:**
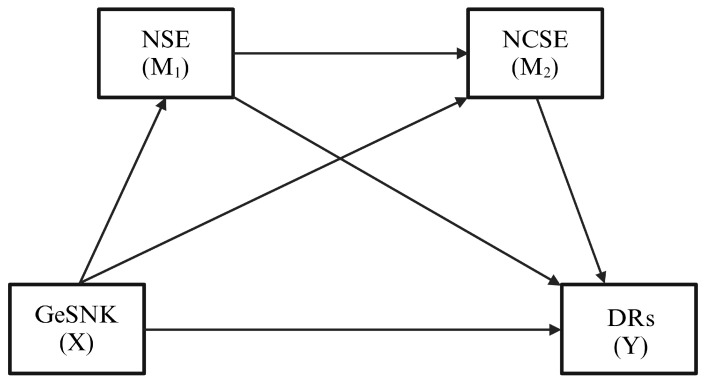
The hypothesis model.

This descriptive, cross-sectional study explored the factors influencing dietary recommendations among CC&Cs by examining the direct and indirect relationships between dietary recommendations, GeSNK, NSE, and NCSE. The study model was constructed using PROCESS Model 6 [33] to assess moderating effects. The hypothesis model is illustrated in Figure 1.

### 2.2. Participants and Procedures

The study included 172 participants (mean age = 28.65 ± 8.46), comprising 70 coach candidates (mean age = 22.89 ± 1.55) enrolled in programs at a university’s Faculty of Sports Sciences in the Marmara region of Turkey and 102 professional coaches (mean age = 32.61 ± 8.98) working in the same region. Ethics approval was obtained from the Acibadem University Scientific Research Ethics Committee (ATADEK-2022/07).

Coach candidates were in the final semester of 4-year programs such as Physical Education, Sports Teaching, and Sports Coaching Education. Although the programs varied in scope and curriculum, graduates could pursue similar careers, provided they met additional requirements and obtained relevant certifications. The coach candidates included in this study had no remaining graduation requirements except for the final examinations in their last-term courses. Data from these participants were collected during the last semester of their 4-year programs, 1 week before their final examinations at their respective faculties.

The coaches who participated in this study had graduated from the aforementioned programs and had accumulated >3 yr of coaching experience. Surveys were administered to these coaches at the amateur sports clubs and facilities where they were employed. All data collection procedures were conducted with the consent of the participants.

Participants self-identified their sports background, with 90 (52.3%) individuals reporting involvement in individual sports (e.g., swimming, tennis, and wrestling) and 82 (47.7%) individuals in team sports (e.g., football, basketball, and volleyball). Descriptive statistics of the participants are presented in Table 1. Additionally, 89.5% of participants indicated that they had taken nutrition courses during their university education.

The required sample size was determined using Green’s formula for regression analysis, N = 50 + 8 m, where m represents the number of predictors [34]. With four predictors, the minimum sample size was 82 participants. A post hoc power analysis conducted using G*Power 3 [35] confirmed that with an α level of 0.05, a sample size of 172, and an effect size of 0.261, the power of the study was 0.999, with a critical F value of 2.425.

### 2.3. Pilot Study

Two scales, the Nutrition Self-Efficacy Scale (NSES) and Nutrition Counseling Self-Efficacy Scale (NCSES), were developed based on an extensive literature review and expert input to evaluate the CC&Cs. A pilot study was conducted to assess the validity and reliability of these scales. Draft versions of the NSES (8 items) and the NCSES (11 items) were administered to 139 students (94 males and 45 females; mean age = 24.44; SD = 1.36) from the Faculty of Sports Sciences. Participants in the pilot study were excluded from the main study.

An exploratory factor analysis (EFA) led to the removal of three items with factor loadings below 0.40 on both scales, and the analysis was repeated. The refined NSES, consisting of five items, was confirmed as a one-factor scale with factor loadings ranging from 0.71 to 0.87, item-scale correlations exceeding 0.30, and a Cronbach’s α of 0.86. Similarly, the NCSES, reduced to eight items, was validated as a one-factor scale with factor loadings between 0.75 and 0.90, item-scale correlations above 0.30, and Cronbach’s α of 0.86 [36]. The detailed EFA and item-total correlation results are presented in Table 2.

### 2.4. Data Collection Tools

Data collection was conducted using the following instruments: the Personal Information Form, NSES, NCSES, and General and Sports Nutrition Knowledge Scale (GeSNK).

#### 2.4.1. Personal Information Form

The Personal Information Form collected demographic and professional data, including age, gender, educational background, coaching experience, and type of sport (individual or team). Additional questions addressed the participants’ source of nutrition information, their exposure to nutrition lessons during their education, and their provision of nutritional recommendations. The responses included both short-answer and multiple-choice items.

#### 2.4.2. Nutrition Self-Efficacy Scale

The NSES is a five-item, 4-point Likert-type scale (e.g., “I can manage my weight even when I don’t exercise”), with response options ranging from strongly agree to strongly disagree, designed to assess participants’ nutrition self-efficacy. As shown in Table 3, the confirmatory factor analysis (CFA) conducted on the NSES data from the current study cohort confirmed the validity of the scale model. The reliability coefficient for the NSES was 0.89. Participants could achieve scores ranging from 5 (minimum) to 20 (maximum) on the scale.

#### 2.4.3. Nutrition Counseling Self-Efficacy Scale

The NCSES is an eight-item 4-point Likert scale (e.g., “I can determine the daily nutritional requirements of the people I coach”), with response options ranging from strongly agree to strongly disagree, designed to assess participants’ nutrition counselling self-efficacy. As shown in Table 3, the CFA results confirmed the NCSES model’s validity in the current study cohort, with a reliability coefficient of 0.95. The possible scores on the scale range from 8 (minimum) to 32 (maximum).

**Table 3:** Confirmatory factor analysis results for NSES and NCSES.

	χ^2^	*df*	χ^2^/*df*	RMSEA	CFI	GFI	NFI
Ideal value **			≤2	≤0.05	0.95 ≤ CFI ≤ 1	0.95 ≤ GFI ≤ 1	0.95 ≤ NFI ≤ 1
Acceptable value **			≤3	≤0.08	0.90 ≤ CFI ≤ 0.95	0.90 ≤ GFI ≤ 0.95	0.90 ≤ NFI ≤ 0.9
NSES	3.057	5	0.611	0.000	1.000	0.993	0.993
NCSES	24.13	14	1.724	0.065	0.991	0.968	0.980

NSES, Nutrition Self-Efficacy Scale; NCSES, Nutrition Counselling Self-Efficacy Scale. ** [36].

#### 2.4.4. General and Sports Nutrition Knowledge Scale

This study used the Turkish-adapted version of the GeSNK Scale, originally developed by Calella et al. [37] and adapted by Altınok and Güvenç [38]. The GeSNK Scale comprises 28 general nutrition (GN) items and 32 sports nutrition (SN) items. Altınok and Güvenç reported reliability coefficients of 0.90 for GN, 0.87 for SN, and 0.92 for the overall scale in the Turkish version. In the present study, the reliability coefficients were 0.86 for GN, 0.79 for SN, and 0.89 for the overall scale.

### 2.5. Statistical Analysis

An exploratory component analysis and item-scale correlation tests were employed in the pilot study to validate the NSES and NCSES. In this study, CFA was used to validate these scales, and Cronbach’s α coefficients assessed reliability. Descriptive statistics were calculated, and the Kolmogorov–Smirnov test determined the suitability of parametric tests.

Nonparametric tests were employed for variables related to gender, sport type, and position. Group comparisons used the Mann–Whitney U test, and the effect sizes were calculated using η^2^ and Cohen’s d, interpreted per Cohen’s criteria [39]. Statistical significance was set at *p* < 0.05. Adequate nutritional knowledge was defined as scoring 70% or higher, based on previous research [22].

The confirmatory factor analysis was conducted using Amos 16 software, while other analyses utilized SPSS 22 software. A regression analysis was performed using the PROCESS macro for SPSS (v. 4.2). The PROCESS macro is a nonparametric statistical tool appropriate for situations where the assumption of data normality is violated. It can be applied to both continuous and dichotomous dependent variables. This macro computes the direct, indirect, and total effects of independent variables on dependent variables. In the present study, the serial multiple mediator model (Model 6) from Hayes [33] was employed to examine the direct and indirect effects of GeSNK on dietary recommendations. The findings suggest that GeSNK influences NSE, which subsequently impacts NCSE and, ultimately, dietary recommendations.

## 3. Results

### 3.1. Descriptive Analysis

Descriptive statistics for the NSES, the NCSES, and GeSNK are presented in Table 4. Regarding GeSNK, 36% of participants scored adequately on GN, 38.4% on SN, and 36.6% on the overall scale. For dietary recommendations, 39.5% of participants frequently made dietary recommendations, while 54.1% occasionally made recommendations. The primary sources of nutrition knowledge were identified as schools (35.5%), trainers (22.7%), families (18.6%), and nutritionists or health professionals (15.1%).

### 3.2. NSES, NCSES, and GeSNK Scale by Gender, Sport Type, and Position

Table 5 illustrates the self-efficacy scores by gender. No significant differences were observed in NSE (*p* > 0.05) or NCSE (*p* > 0.05) between genders. Similarly, GeSNK scores by gender showed no significant differences in GN (*p* > 0.05) or the overall scale (*p* > 0.05). However, significant differences were noted in SN scores (*p* < 0.05), with male participants demonstrating greater sports nutrition knowledge. The effect size for SN was medium to small (d = 0.418).

Participants in individual sports exhibited significantly higher NSE (*p* < 0.05) and NCSE (*p* < 0.05) scores than those in team sports, with effect sizes of medium to small (d = 0.408) for NSE and medium (d = 0.497) for NCSE. No significant differences in GeSNK scores were observed between sport types (*p* > 0.05). Detailed comparisons by sport type are shown in Table 6. Table 7 shows no significant differences in NSE, NCSE, or GeSNK scores based on participants’ positions among the CC&Cs (*p* > 0.05).

### 3.3. Relationships Between NSE, NCSE, GeSNK Scale, and Dietary Recommendations

Table 8 summarizes the regression analysis for dietary recommendations in CC&Cs. GeSNK significantly affected NSE (a^1^ = 0.0610; *p* < 0.05), though the explained variance was low (R^2^ = 0.051), indicating a limited but statistically significant effect (*p* < 0.05).

GeSNK and NSE both significantly influenced NCSE (F(2,169) = 59.07; *p* < 0.05). While NSE had a direct significant effect on NCSE (d^1^ = 0.9729; *p* < 0.05), GeSNK did not (a^2^ = 0.0178; *p* > 0.05), suggesting that GeSNK impacts NCSE indirectly via NSE, accounting for 41% of NCSE variance. GeSNK, NSE, and NCSE significantly influenced dietary recommendations (F(3.168) = 16.9402; *p* < 0.05), collectively explaining 23% of its variance. The direct effect of NCSE on dietary recommendations was significant (b^2^ = 0.0394; *p* < 0.05), but neither GeSNK (c^1^ = 0.0063; *p* > 0.05) nor NSE (b^1^ = 0.0214; *p* > 0.05) had a direct effect. Thus, GeSNK indirectly influenced dietary recommendations through other pathways.

The indirect paths GeSNK → NSE → DR and GeSNK → NCSE → DR were not significant, as the bootstrap confidence intervals included 0. This shows that GeSNK does not significantly affect dietary recommendations through NCSE or NSE. However, the GeSNK → NSE → NCSE → DR pathway (a^1^d^1^b^2^ = 0.0023) had a significant and positive impact, as the bootstrap confidence interval was above 0. This finding suggests that GeSNK influences dietary recommendations indirectly by first impacting NSE, which subsequently affects NCSE.

**Figure 2:**
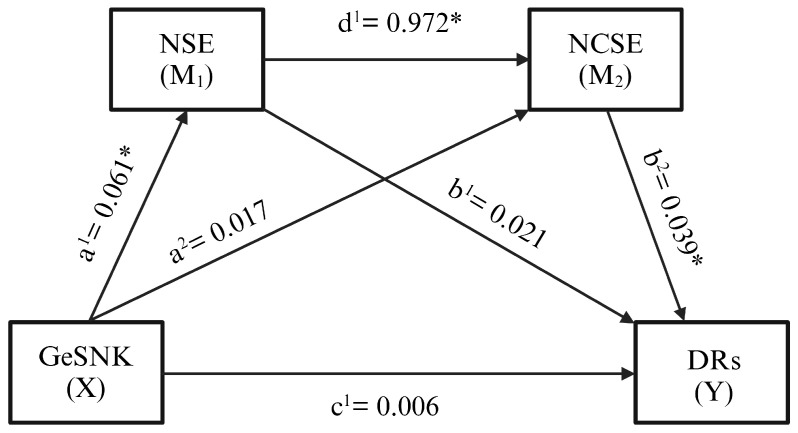
The test results of the hypothesis model. * *p* < 0.05.

Furthermore, the total effect of the serial multiple mediator model (C = c′ + a^1^b^1^ + a^2^b^2^ + a^1^d^1^b^2^) of GeSNK (X) on dietary recommendations (Y) was significant (C = 0.0106; *p* < 0.05). This analysis confirms that GeSNK exerts an indirect significant impact on dietary recommendations, with no direct effect. The hypothesis model results are presented in Figure 2.

## 4. Discussion

This study aimed to explore the factors influencing dietary recommendations among CC&Cs by examining the direct and indirect relationships between dietary recommendations, GeSNK, NSE, and NCSE.

The results demonstrate that GeSNK indirectly affects dietary recommendations by influencing NSE and NCSE, but it does not directly impact dietary recommendations. These findings align with previous research showing that dietary recommendations are not directly affected by nutritional knowledge [14].

Consistent with earlier studies reporting insufficient nutritional knowledge among coaches [1,11,14,20,23], a significant proportion of participants lacked adequate GN knowledge (64%) and SN knowledge (61.6%). Coaches play a critical role in shaping athletes’ nutritional behavior and knowledge [1,5,6,7,8,9,11,23], highlighting the importance of targeted nutritional education for coaches [1,21,22]. In this study, 35.5% of participants identified schools as their primary source of nutritional knowledge, suggesting a need to revise sports science curricula to include more nutrition-focused courses. Moreover, 22.7% cited former coaches as a knowledge source, emphasizing the value of integrating nutritional training into coach education programs.

General nutritional knowledge among CC&Cs did not significantly differ based on gender or sport type, a finding consistent with prior studies reporting no significant differences in nutritional knowledge across demographics such as age, gender, educational level, coaching level, or sport type [9,10,22].

The results revealed no significant differences in CC&Cs’ nutritional knowledge. Consistent with previous studies, the duration of coaching experience did not influence nutritional knowledge [18,21]. Interestingly, the most experienced coaches demonstrated the lowest nutritional knowledge scores [22].

The research found that GeSNK exerted a minor influence on NSE. NSE, a critical predictor of nutritional behavior, is highly significant. Previous studies have shown that interventions targeting NSE, such as nutrition programs [31], educational initiatives [40], and behavior-change strategies [41], positively impact athletes. Similar approaches tailored for coaches should aim to enhance their NSE, enabling them to model healthier behavior for athletes by emphasizing the importance of proper nutrition.

Among the variables examined, NCSE was the only one with a direct impact on dietary recommendations. However, NCSE was influenced by GeSNK not directly but rather through NSE. Together, GeSNK and NSE accounted for 41% of the variation in NCSE. Other variables influencing NCSE among CC&Cs warrant further investigation. Within the framework of social cognitive theory, it can be hypothesized that coaches’ NCSE originates from their observations of former coaches.

The findings revealed that participants involved in individual sports exhibited more favorable NSE and NCSE scores compared with those in team sports. This could be attributed to the closer coach–athlete relationships in individual sports [42], which might enhance the modeling process [25]. However, if future research confirms that modeling is the primary source of coaches’ NCSE, the practical implications would remain limited, as modeling is an inherent part of coaching. Consequently, coaches may inadvertently transfer their NSE and GeSNK to athletes, and when these athletes transition to coaching roles, their NCSE may reflect this inherited influence.

Remarkably, only 6.4% of participants reported not providing dietary recommendations to athletes. Even coach candidates appeared to offer dietary recommendations during professional practice courses, which are part of their training programs. This highlights the need to consider factors beyond the study’s independent variables. While self-efficacy is regarded as the primary personal factor in Bandura’s theory, outcome expectancy also constitutes a significant motivational element. Individuals behave in manners they perceive will yield positive consequences and seek guidance from models they consider capable of imparting essential abilities. The dietary recommendations of coaches may be related to outcome expectation [25,26]. Based on SCT, environmental factors such as performance pressures from parents and administrators may compel coaches to provide dietary recommendations and encourage them to monitor athletes’ nutrition and body composition closely [23]. Furthermore, athletes often feel comfortable receiving dietary recommendations from coaches [1,3].

Despite these findings, there are concerns about the potential adverse health effects of improper dietary recommendations [23]. To address these concerns, collaboration between nutritionists and coaches is essential to ensure that athlete health and performance are supported effectively [3,43]. In cases where access to a dietitian is available, coaches can be informed with justification about the nutritional arrangements made for their athletes. In contexts where access to a dietitian is limited, such as high school sports teams, organizational arrangements can be made to facilitate coaches’ access to dietitians. Coaches can be provided with dietitian support regarding their athletes’ nutrition individually or through focus group meetings.

## 5. Conclusions

The findings highlight inadequate nutritional knowledge among CC&Cs, with only a minority abstaining from providing dietary recommendations. Notably, NCSE was found to directly influence dietary recommendations. Identifying additional variables that explain NCSE and dietary recommendations is crucial.

Continuing to provide dietary recommendations despite knowledge gaps may jeopardize athletes’ health. This underscores the urgent need for effective education programs and regular professional development initiatives to equip coaches with reliable and comprehensive nutrition knowledge. Coaches and other sports personnel who have contact with athletes on the subject of nutrition should be aware that the science of nutrition is constantly evolving and should accept increasing their nutritional knowledge as a part of their professional development.

### Limitations

This study provides valuable insights into the factors influencing coaches’ diet recommendations but also has several limitations. First, diet recommendations were evaluated solely in terms of frequency, with no assessment of their content or quality. Additionally, GeSNK was not analyzed in detail for specific food categories or micronutrients, and both NSE and NCSE were discussed broadly. Future studies should investigate the relationships between specific dietary recommendations, nutritional knowledge, NSE, and NCSE. Although no significant differences were found between the GeSNK, NCSE, and NSE of coaches and coach candidates, this can be seen as another limitation of the study, and it can be suggested that the study be repeated on a participant group consisting of only experienced coaches. Finally, this study did not collect data from athletes or assess the impact of coaches’ GeSNK, NSE, NCSE, or dietary recommendations on athletes. These gaps warrant further research to provide a comprehensive understanding of these dynamics.

## Figures and Tables

**Table 1:** Participant characteristics of the study.

Variable		*n* (%)
Gender	Male	111 (64.5)
	Female	61 (35.5)
Position	Coach Candidates	70 (40.7)
	Coach	102 (59.3)
Sport Type	Individual	90 (52.3)
	Team	82 (47.7)
Source of NK	Family	32 (18.6)
	School	61 (35.5)
	Media	14 (8.1)
	Coach	39 (22.7)
	HP	26 (15.1)

NK, nutrition knowledge; HP, health professional; *n*, number.

**Table 2:** EFA and item-total results for NSES and NCSES.

	NSES		NCSES	
Item No	Factor Loading	Item-Total Correlation	Factor Loading	Item-Total Correlation
1	0.848	0.737	0.846	0.794
2	0.741	0.602	0.902	0.867
3	0.867	0.760	0.878	0.837
4	0.835	0.712	0.891	0.850
5	0.705	0.560	0.836	0.781
6			0.753	0.687
7			0.863	0.817
8			0.858	0.807
Eigenvalues	3.22		5.84	
Explained Variance %	64.31		%73.02	
Cronbach Alpha α	0.86		0.95	

NSES, Nutrition Self-Efficacy Scale; NCSES, Nutrition Counselling Self-Efficacy Scale.

**Table 4:** Descriptive statistics for NSE, NCSE, and GeSNK.

Variable		f (%)/Median; IQR
NSE		15.0; 4.75
NCSE		23.0; 5
GeSNK	GN	42.0; 13
	SN	21.0; 6
	GeSNK Whole	62.0; 18
Dietary Recommendations	Often	68 (39.5)
	Occasionally	93 (54.1)
	Never	11 (6.4)
Source of NK	Family	32 (18.6)
	School	61 (35.5)
	Media	14 (8.1)
	Coach	39 (22.7)
	HP	26 (15.1)

GeSNK, general and sports nutrition knowledge; GN, general nutrition; SN, sports nutrition; HP, health professional.

**Table 5:** Comparison of NSE, NCSE, and GeSNK in terms of gender.

Variable	Gender	*n*	Median	IQR	U **	*p*	*η* ^2^	d_Cohen_
NSE	Male	111	15.0	5.0	3023.0	0.239	0.008	0.178
	Female	61	15.0	2.5				
NCSE	Male	111	24.0	5.0	2792.5	0.056	0.021	0.293
	Female	61	23.0	4.0				
GN	Male	111	42.0	13.0	3359.5	0.934	0	0.013
	Female	61	42.0	14.5				
SN	Male	111	22.0	5.0	2546.5	0.007 *	0.042	0.418
	Female	61	20.0	7.0				
GeSNK Whole	Male	111	62.0	16.0	3029.0	0.254	0.008	0.175
	Female	61	61.0	19.5				

GeSNK, general and sports nutrition knowledge; GN, general nutrition; SN, sports nutrition; NSE, nutrition self-efficacy; NCSE, nutrition counselling self-efficacy. * *p* < 0,05; ** Mann–Whitney U.

**Table 6:** Comparison of NSE, NCSE, and GeSNK in terms of sport type.

Variable	Sport Type	*n*	Median	IQR	u **	*p*	*η* ^2^	d_Cohen_
NSE	Individual	90	15.0	4.00	2834	0.008 *	0.04	0.408
	Team	82	14.0	3.25				
NCSE	Individual	90	24.0	4.00	2657.5	0.001 *	0.058	0.497
	Team	82	22.0	5.00				
GN	Individual	90	42.5	12.00	3562.5	0.696	0.001	0.06
	Team	82	41.0	14.00				
SN	Individual	90	22.0	8.00	3593.5	0.767	0.001	0.045
	Team	82	21.0	5.00				
GeSNK Whole	Individual	90	62.0	18.50	3640	0.878	0	0.023
	Team	82	61.0	17.25				

GeSNK, general and sports nutrition knowledge; GN, general nutrition; SN, sports nutrition; NSE, nutrition self-efficacy; NCSE, nutrition counselling self-efficacy. * *p* < 0.05; ** Mann–Whitney U.

**Table 7:** Comparison of NSE, NCSE, and GeSNK in terms of position.

Variable	Position	*n*	Median	IQR	u *	*p*	*η* ^2^	d_Cohen_
NSE	CC	70	15.0	5.00	3546.5	0.941	0	0.011
	C	102	15.0	4.00				
NCSE	CC	70	24.0	5.25	3131.5	0.168	0.011	0.21
	C	102	23.0	4.00				
GN	CC	70	39.5	13.75	2947.0	0.052	0.022	0.299
	C	102	43.0	11.25				
SN	CC	70	21.0	6.50	3305.5	0.408	0.004	0.126
	C	102	22.0	5.00				
GeSNK Whole	CC	70	61.0	19.50	3034.5	0.095	0.016	0.257
	C	102	64.5	14.50				

GeSNK, general and sports nutrition knowledge; GN, general nutrition; SN, sports nutrition; NSE, nutrition self-efficacy; NCSE, nutrition counselling self-efficacy. * Mann–Whitney U.

**Table 8:** Regression coefficients, standard errors, and model summary for dietary recommendations by CC&Cs.

	Antecedent					Consequent								95% CI **	
			M1 (NSE)				M2 (NCSE)				Y (DR)				
Effect			Coeff	SE	*p*		Coeff	SE	*p*		Coeff	SE	*p*	LLCI	ULCI
Direct	X (GeSNK)	a^1^	0.0610	0.0202	0.0029 *	a^2^	0.0178	0.0253	0.4822	c^1^	0.0063	0.0033	0.0507		
	M1 (NSE)		-	-	-	d^1^	0.9729	0.0935	0.0000 *	b^1^	0.0214	0.0156	0.1711		
	M2 (NCSE)		-	-	-		-	-	-	b^2^	0.0394	0.0100	0.0001 *		
	Constant		11.1943	1.2574	0.0000		7.1790	1.8558	0.0002		0.7297	0.2517	0.0042		
			R^2^ = 0.0510				R^2^ = 0.4115				R^2^ = 0.2322				
			F(1.170) = 9.1407; *p* = 0.0029				F(2.169) = 59.0734; *p* = 0.0000				F(3.168) = 16.9402, *p* = 0.0000				
Indirect	GeSNK → NSE → DR					a^1^ b^1^	0.0013	0.0020						−0.0007	0.0040
	GeSNK → NCSE → DR					a^2^ b^2^	0.0007	0.0011						−0.0013	0.0029
	GeSNK → NSE → NCSE → DR					a^1^ d^1^ b^2^	0.0023	0.0011						0.0006	0.0051
Total						C	0.0106	0.0035	0.003 *						

* *p* < 0.05; ** Bootstrap.

## Data Availability

The raw data supporting the conclusions of this article will be made available by authors on request.

## Abbreviations

The following abbreviations are used in this manuscript:

CC&Cs	Coach candidates and coaches
SCT	Social cognitive theory
CFA	Confirmatory factor analysis
DRs	Dietary recommendations
EFA	Exploratory factor analysis
GeSNK	General and sports nutrition knowledge
GN	General nutrition
NCSE	Nutrition counseling self-efficacy
NCSES	Nutrition Counseling Self-Efficacy Scale
NSE	Nutrition self-efficacy
NSES	Nutrition Self-Efficacy Scale
SN	Sports nutrition
